# Influenza Illness and Hospitalizations Averted by Influenza Vaccination in the United States, 2005–2011

**DOI:** 10.1371/journal.pone.0066312

**Published:** 2013-06-19

**Authors:** Deliana Kostova, Carrie Reed, Lyn Finelli, Po-Yung Cheng, Paul M. Gargiullo, David K. Shay, James A. Singleton, Martin I. Meltzer, Peng-jun Lu, Joseph S. Bresee

**Affiliations:** 1 Influenza Division, National Center for Immunizations and Respiratory Diseases, Centers for Disease Control and Prevention, Atlanta, Georgia, United States of America; 2 Immunizations Services Division, Centers for Disease Control and Prevention, Atlanta, Georgia, United States of America; 3 Division of Preparedness and Emerging Infections, National Center for Enteric and Zoonotic Infectious Disease, Centers for Disease Control and Prevention, Atlanta, Georgia, United States of America; Harvard School of Public Health, United States of America

## Abstract

**Context:**

The goal of influenza vaccination programs is to reduce influenza-associated disease outcomes. Therefore, estimating the reduced burden of influenza as a result of vaccination over time and by age group would allow for a clear understanding of the value of influenza vaccines in the US, and of areas where improvements could lead to greatest benefits.

**Objective:**

To estimate the direct effect of influenza vaccination in the US in terms of averted number of cases, medically-attended cases, and hospitalizations over six recent influenza seasons.

**Design:**

Using existing surveillance data, we present a method for assessing the impact of influenza vaccination where impact is defined as either the number of averted outcomes or as the prevented disease fraction (the number of cases estimated to have been averted relative to the number of cases that would have occurred in the absence of vaccination).

**Results:**

We estimated that during our 6-year study period, the number of influenza illnesses averted by vaccination ranged from a low of approximately 1.1 million (95% confidence interval (CI) 0.6–1.7 million) during the 2006–2007 season to a high of 5 million (CI 2.9–8.6 million) during the 2010–2011 season while the number of averted hospitalizations ranged from a low of 7,700 (CI 3,700–14,100) in 2009–2010 to a high of 40,400 (CI 20,800–73,000) in 2010–2011. Prevented fractions varied across age groups and over time. The highest prevented fraction in the study period was observed in 2010–2011, reflecting the post-pandemic expansion of vaccination coverage.

**Conclusions:**

Influenza vaccination programs in the US produce a substantial health benefit in terms of averted cases, clinic visits and hospitalizations. Our results underscore the potential for additional disease prevention through increased vaccination coverage, particularly among nonelderly adults, and increased vaccine effectiveness, particularly among the elderly.

## Introduction

Since 2010, all persons 6 months of age and older in the United States have been recommended to receive annual influenza vaccination, making the U.S the only country with universal influenza vaccine recommendations [1]. The recommendation for universal vaccination was made after more than a decade of incremental expansions in the recommendations to include individuals at high risk for severe influenza or influenza-associated complications, or those who might spread infection to high-risk persons [2]. The health and economic benefits of influenza vaccination have traditionally been evaluated using vaccine coverage surveys, by observational studies of vaccine effectiveness or clinical trials of vaccine efficacy in specific populations [3]–[13], or by cost-effectiveness studies of influenza vaccination in certain groups of people or at certain times, based on data from sporadic studies [14]–[20]. These studies have been valuable tools in establishing the potential impact of influenza vaccines in specific populations or to monitor program operations. However, no studies have provided a mechanism for the replicable assessment of national numbers of cases and hospitalizations that are prevented by vaccination each year.

Since the ultimate justification for influenza vaccination programs is that they reduce influenza-associated disease outcomes, it is desirable to have an approach that describes with annual regularity the reduced national burden of influenza during each influenza season. Such an approach would allow for a clear understanding of the value of influenza vaccination in the U.S. and would enable us to trace vaccine program performance over time and within demographic groups. Such data would also identify areas where improvements would lead to greatest benefits. In this paper, we present a national model for estimating the annual direct impact of influenza vaccination in terms of averted morbidity in order to better understand the impact of influenza immunization programs in the United States and the factors that drive this impact. This method of estimation can be updated annually, and can be used by public health professionals to estimate and/or project averted burden using assumptions of vaccination coverage, effectiveness, and influenza clinical attack rates. These data can easily be communicated to stakeholders concerning the value of influenza vaccines.

## Methods and Data

### 1. Methods Overview

#### 1.1 Estimating averted burden

In this paper, burden is defined in terms of influenza cases, medically attended illnesses, and hospitalizations (henceforth referred to as “outcomes”). We estimated the averted burden of influenza-related outcomes in several steps. First, we estimated the number of outcomes that occurred in each season using existing national surveillance data as a primary input. We then computed intermediary inputs such as the rates of influenza illness and influenza hospitalization among susceptible individuals for each month of each season while accounting for vaccination coverage, vaccine efficacy, and disease occurrence, and we used these rates to project the burden of influenza that would have occurred in the absence of vaccination. The difference between the estimated number of outcomes with vs. without vaccination equals the burden averted by vaccination. Each season’s model was stratified by month to accommodate time-sensitive patterns of vaccination coverage and disease occurrence, and was built to reflect the length of each season –7 months for seasons 2005–2006 through 2008–2009 and 2010–2011 (October through April), and 12 months during the 2009 H1N1 pandemic (May 2009 through April 2010). Averted burden was estimated for four age categories: 6 months-4 years old, 5–19 years old, 20–64 years old, and 65 years and older. This approach only estimates the direct effects of vaccination to vaccinated persons, and does not reflect how the underlying attack rates and disease transmission patterns would have changed in the complete absence of vaccination; the indirect impact of vaccination (the additional outcomes that may have been averted by decreasing the overall disease infectivity through vaccination) is therefore excluded from the estimates. A detailed methods description is available in Appendix S1.

Confidence intervals for the reported results were estimated using a Monte Carlo algorithm, drawing values from sampling distribution of the input variables used in the model. Additional details about these sampling distributions are given in Appendix S1.

#### 1.2 Estimating the prevented fraction

While the number of averted outcomes each season depends directly on vaccination coverage and vaccine effectiveness during that season, it also depends on the influenza attack rate – i.e., seasons with high attack rates will result in a higher number of averted outcomes assuming the same rate of vaccination coverage. Therefore, we present averted influenza burden not only in terms of absolute numbers, but also in terms of the prevented fraction. We define the prevented fraction as the proportion of averted outcomes out of potential outcomes in the absence of vaccination. During each influenza season, the prevented fraction is the same across outcomes (cases, medically-attended cases, and hospitalizations) because the number of cases and MA cases were calculated as proportions of the estimated number of hospitalizations each season.

### 2. Data Sources

#### 2.1 Rates of influenza-associated hospitalization

We used data on the rates of reported influenza-associated hospitalizations from CDC’s Emerging Infections Program (EIP) to estimate the annual number of influenza hospitalizations and illnesses from 2005 to 2011 (both summarized in Table 1). Although total annual estimates of influenza hospitalizations have been reported in the literature for multiple seasons prior to 2001 [21], the EIP is the only data source that provides timely real-time tracking of influenza hospitalizations in the U.S. as each season progresses, allowing us to reconstruct the baseline shapes of recent seasons’ epidemiological curves. The EIP conducts surveillance for laboratory-confirmed influenza-related hospitalizations in both children and adults in 60 counties covering 12 metropolitan areas of 10 states (approximately 22 million people). Using the EIP’s unique aspect of month-specific and age-specific hospitalization reporting, we obtained estimates of the reported rates of influenza hospitalization across 4 age groups for the six most recent seasons with complete EIP data.

**Table 1:** Summary of input variables, by age group (95% confidence intervals in parentheses).

Age	Influenza Season	CumulativeVaccineCoverage (%)^1^	VaccineEffectiveness (%)^2^	Total Population^3^	Cumulative Hospitalization Rate (per 100,000)^4^	Estimated number of hospitalizations^5^	Estimated number of cases^6^	Estimated number of medically-attended cases^7^
0–4	‘05–06	34.9 (30.5–40.1)	42.1 (18.5–58.9)	18,435,382	26.7	13,466 (8,314–21,733)	1,931,620 (1,192,528–3,117,420)	811,280 (500,745–1,358,860)
0–4	‘06–07	44.5 (39.5–50.4)	50.5 (41.7–57.9)	18,551,515	19.7	9,992 (6,048–16,331)	1,433,221 (867,572–2,342,474)	601,953 (367,384–1,016,260)
0–4	‘07–08	48.2 (42.5–54.9)	47.3 (39.7–54.0)	18,829,160	29.5	15,197 (9,485–24,420)	2,179,852 (1,360,507–3,502,832)	915,538 (573,725–1,523,857)
0–4	‘08–09	51.0 (44.3–58.9)	50.5 (43.2–56.9)	19,037,307	27.8	14,505 (9,084–23,240)	2,080,606 (1,303,035–3,333,529)	873,855 (550,950–1,452,562)
0–4	‘09–10	41.2 (36.0–47.2)	61.7 (24.9–80.5)	19,169,690	71.7	37,671 (24,339–59,277)	5,403,551 (3,491,158–8,502,690)	3,620,379 (2,337,447–5,702,209)
0–4	‘10–11	59.6 (53.8–66.7)	68.0 (53.0–78.0)	18,060,883	30.7	15,203 (9,577–24,342)	2,180,689 (1,373,671–3,491,566)	915,889 (578,337–1,519,444)
5–19	‘05–06	15.0 (13.4–16.9)	42.1 (18.5–58.9)	61,588,987	5.0	8,501 (5,059–14,050)	3,100,575 (1,845,063–5,124,334)	1,302,241 (777,957–2,231,660)
5–19	‘06–07	20.1 (18.0–22.5)	50.5 (41.7–57.9)	61,777,006	3.3	5,622 (3,060–9,763)	2,050,560 (1,115,886–3,560,631)	861,235 (471,528–1,543,049)
5–19	‘07–08	23.3 (21.1–25.9)	47.3 (39.7–54.0)	61,935,681	5.8	9,856 (6,083–16,094)	3,594,664 (2,218,402–5,869,773)	1,509,759 (935,087–2,559,000)
5–19	‘08–09	28.2 (25.2–31.6)	50.5 (43.2–56.9)	62,045,041	6.5	11,020 (6,880–17,884)	4,019,214 (2,509,319–6,522,328)	1,688,070 (1,059,519–2,846,351)
5–19	‘09–10	30.6 (27.9–33.6)	61.7 (24.9–80.5)	62,121,035	32.9	56,035 (36,964–86,594)	20,436,537 (13,481,260–31,581,548)	10,422,634 (6,848,327–16,160,233)
5–19	‘10–11	38.2 (35.1–41.6)	61.0 (47.0–71.0)	63,066,194	8.1	14,000 (8,723–22,537)	5,105,885 (3,181,547–8,219,579)	2,144,472 (1,342,665–3,577,399)
20–64	‘05–06	20.4 (19.1–21.5)	42.1 (18.5–58.9)	176,976,709	4.6	22,276 (14,278–35,267)	3,301,243 (2,115,936–5,226,613)	1,386,522 (890,318–2,280,009)
20–64	‘06–07	23.3 (21.9–24.8)	50.5 (41.7–57.9)	178,997,496	3.1	14,972 (9,397–23,878)	2,218,860 (1,392,616–3,538,704)	931,921 (591,237–1,545,199)
20–64	‘07–08	26.2 (24.8–27.8)	47.3 (39.7–54.0)	180,855,780	9.7	14,972 (32,208–73,786)	2,218,860 (4,773,186–10,935,062)	931,921 (2,007,674–4,766,405)
20–64	‘08–09	28.7 (26.8–30.8)	50.5 (43.2–56.9)	182,377,351	4.2	21,055 (13,580–33,318)	3,120,343 (2,012,580–4,937,763)	1,310,544 (848,358–2,158,912)
20–64	‘09–10	18.5 (17.2–20.0)	61.7 (24.9–80.5)	184,015,269	32.2	162,213 (110,968–245,921)	24,040,015 (16,445,394–36,445,430)	8,894,806 (6,053,895–13,513,431)
20–64	‘10–11	31.4 (29.7–33.3)	50.0 (35.0–61.0)	185,209,998	13.7	69,715 (47,349–105,765)	10,331,754 (7,017,050–15,674,409)	4,339,337 (2,959,542–6,861,096)
65+	‘05–06	66.0 (62.5–69.9)	29.5 (13.0–41.2)	36,703,697	40.1	40,307 (26,558–62,423)	2,606,654 (1,717,526–4,036,920)	1,094,795 (721,574–1,754,457)
65+	‘06–07	67.7 (64.1–71.6)	35.4 (29.2–40.5)	37,205,916	16.4	16,715 (10,370–26,814)	1,080,990 (670,632–1,734,089)	454,016 (282,873–754,991)
65+	‘07–08	69.2 (65.8–72.8)	33.1 (27.8–37.8)	37,867,145	75.9	78,718 (53,179–119,812)	5,090,700 (3,439,065–7,748,262)	2,138,094 (1,449,362–3,396,737)
65+	‘08–09	69.6 (65.5–74.0)	35.4 (30.2–39.8)	38,799,891	14.2	15,106 (9,310–24,539)	976,894 (602,097–1,586,945)	410,295 (253,636–691,387)
65+	‘09–10	26.6 (23.6–30.0)	43.2 (17.4–56.4)	39,570,590	31.6	34,257 (21,834–54,531)	2,215,423 (1,412,020–3,526,515)	1,240,637 (788,534–1,980,071)
65+	‘10–11	69.7 (65.7–74.0)	36.0 (0.0–66.0)	40,267,984	64.3	70,938 (47,937–107,624)	4,587,560 (3,100,113–6,960,043)	1,926,775 (1,301,645–3,050,473)
All ages season average						128,719 (88,431–208,324)	19,217,711 (13,106,361–31,253,243)	8,454,495 (5,748,720–14,040,675)

1 Source: National Health Interview Survey (NHIS), United States, 2006–2011. The season-cumulative vaccine coverage rates in this table are for summary description only; model employs incremental monthly age-specific values. Estimates of the cumulative monthly proportion vaccinated through end of April of each season were developed using the Kaplan-Meier product limit method for receipt of most recent reported influenza vaccination; estimates based on the NHIS may differ from estimates from other data sources (http://www.cdc.gov/flu/professionals/vaccination/vaccinecoverage.htm).

2 Estimates for seasons 05/06 - 08/09 represent combined estimates from available studies each season and were calculated as averages weighted by the inverse variance of each study. Sources: 2005/06 season: Belongia 2009, Ohmit 2008, Skowronski 2007. 2006/07 season: Belongia 2009, Skowronski 2009. 2007/08 season: Monto 2009, Belongia 2011, Frey 2010. 2008/09 season: Skowronski 2010, Shay 2011. 2009/10 pandemic season: Griffin et al. 2011. 2010/11 season: Treanor et al. 2012. VE estimates for the 2010/11 season are age-specific. VE estimates for seasons 2005/06-2009/10 are not age-specific, except for a downward adjustment applied to the 65+ age group as follows: VE for the 65+ age group is assumed to be 70% of the VE for the younger age groups.

3 Source: U.S. Census Bureau, Population Division. Annual Estimates of the Resident Population by Sex and Five-Year Age Groups for the United States.

4 Source: Centers for Disease Control and Prevention Emerging Infections Program (EIP) 2005–2010. The season-cumulative EIP hospitalization rates in this table are for summary description only; model employs month-specific and age-specific values.

5 Estimated using EIP hospitalization rates adjusted for underreporting. The underreporting adjustment multiplier was obtained from Reed 2009 and presumed constant across seasons and age categories at 2.7(CI 1.7–4.5).

6 Based on the estimated number of hospitalizations and age-specific case-hospitalization ratios from Reed 2009.

7 Based on the estimated number of cases and medically-attended (MA) ratios. The MA ratios used for the 2009/10 season are age-specific; source: Biggerstaff 2012. Age-specific and season-specific MA ratios for the five preceding seasons (2005/06–2009/10) were not available and were presumed constant at 42.0%(CI 37.9%–48.5%); source: Kamimoto 2010.

Since EIP records only hospitalizations confirmed by influenza laboratory test, we needed to account for underreporting. Underreporting occurs when a person truly hospitalized with influenza is not tested for influenza, or when the test does not detect influenza due to the sensitivity of the type of test used or its timing. To account for underreporting, we adjusted the EIP estimate by applying a hospitalization underreporting multiplier. Reed et al. (2009) estimated that during the 2009 H1N1 pandemic, every reported influenza hospitalization represented 2.7 total hospitalizations (95% confidence interval (CI) 1.7–4.5) [22]. Although no estimates of the hospitalization underreporting multiplier were available for seasonal influenza prior to 2009, recent evidence indicates that the disparity in hospitalization underreporting rates between pandemic and non-pandemic seasons was not very substantial [23]. We assumed that hospitalization under-ascertainment in the EIP system before the 2009 pandemic occurred at the same rate as it did during the 2009 pandemic, and adjusted the reported EIP hospitalization rates accordingly by a factor of 2.7 (CI 1.7–4.5) for all seasons.

The estimated number of influenza-associated hospitalizations derived in this study is comparable to previously reported estimates for earlier seasons. Thompson et al (2004) estimated that during 1979–2001 the average seasonal number of influenza-related pneumonia and influenza hospitalizations was 133,900 (CI 30,757–271,529). In this study, the estimated average seasonal number of influenza hospitalizations during 2005–2011 is 128,719 (CI 88,431–208,324) (Table 1). Although these estimates were obtained through different methodologies and for different time periods, their similarity illustrates the relative consistency in the results of the respective estimation approaches.

#### 2.2 Rates of influenza illness

We estimated the number of influenza illnesses in the US population by applying a ratio of cases to hospitalizations to the number of estimated influenza-associated hospitalizations. The case-hospitalization ratio is based on data from the 2009 season as described by Reed et al. (2009) and varies by age group (Table 1).

#### 2.3 Rate of Medically-Attended (MA) illness

Only a fraction of persons with influenza illness seek medical care. This fraction was estimated from two sources, depending on the season. For the 2009 H1N1 pandemic, we used age-specific estimates of the percentage of persons with influenza-like illness (ILI) who reported receiving medical attention for their illness [24]. For all other seasons, we used data from an earlier study which found that 42% (CI 37.9%–48.5%) of persons with ILI sought medical care during the 2006–2007 influenza season [25]. Although this pre-pandemic estimate is not age-specific and is based on a single season, it is the only such data point available before the 2009 pandemic. Because of the possible differences in public awareness and health-seeking behavior during the pandemic, we apply this estimate to the five non-pandemic seasons.

#### 2.4 Influenza vaccination coverage

We defined the monthly incremental vaccination coverage (IVC) for influenza as the proportion of the population that received influenza vaccination in each month. Monthly IVC estimates by age group were obtained from the National Health Interview Survey (NHIS) (Table 1).

#### 2.5 Influenza vaccine effectiveness

Vaccine effectiveness (VE) is the percentage reduction in risk of influenza illness that is attributable to vaccination. Seasonal VE is estimated through clinical trials or observational studies; since estimates of vaccine effectiveness can vary across different studies for the same season, we used an aggregate annual VE estimate based on the range of available VE estimates in the literature for each season [3]–[13] by averaging the range of available values and weighting by the inverse of the variance for each study. For all but the most recent season in this study, VE estimates that were specific to each age group were not available. However, there is evidence that the immunogenic response to the influenza vaccine is decreased among persons 65 years and older [26]–[31]. To reflect this, we made the assumption that VE in the elderly population was 70% of the VE estimates available for younger age groups (those under 65 years) during seasons when age-specific VE estimates were not available (2005–2006 through 2009–2010). To further reflect the uncertainty of this assumption we performed sensitivity analyses where the VE among persons 65 and older was assumed to be, alternatively, 40% or 80% of the VE among younger populations.

## Results

Influenza vaccination averted approximately 13.6 (CI 8.0–22.8) million illnesses, 5.8 (CI 3.4–10.1) million medical visits, and 112,900 (CI 65,000–191,500) influenza-related hospitalizations during the 6-year period (Tables 2, 3, 4). The largest number of averted cases occurred during the most recent season of the study period, 2010–2011, when 5.0 (CI 2.9–8.6) million influenza cases, 2.1 (1.2–3.7) million medical visits, and 40,400 (CI 20,800–73,100) hospitalizations were prevented by vaccination, corresponding to a prevented fraction of 18.5% (CI 16.0%–20.3%). The season with the lowest number of averted outcomes was 2006–2007, when approximately 1.1 (CI 0.6–1.7) million cases were averted. The relatively low number of averted outcomes in 2006–2007 reflects the low intensity of the season, and the fact that fewer cases can be prevented when the underlying incidence is low; indeed, the 2006–2007 season had the lowest attack rate among persons under 65 of all seasons in our study, as shown by the hospitalization rates in Table 1. Despite having the lowest number of averted cases, the 2006–2007 season outperformed the previous season in terms of the prevented fraction, reflecting the season’s improvement in vaccine coverage and vaccine effectiveness (13.5% (CI 13.5%–13.6%) prevented fraction in 2006–2007 vs. 10.6% (CI 9.3%–11.6%) in 2005–2006, Table 2).

**Table 2:** Estimated number of influenza cases averted by vaccination and the associated prevented fraction, 2005/06–2010/11 influenza seasons (95% confidence interval in parentheses).

Age	Influenza season	Number of averted cases		Prevented fraction (%)	
0–4	‘05–06	281,127	(155,540–491,787)	12.7	(11.4–13.8)
0–4	‘06–07	317,922	(190,724–522,177)	18.2	(17.9–18.3)
0–4	‘07–08	478,042	(297,418–769,426)	18	(17.9–18.0)
0–4	‘08–09	535,981	(334,699–858,070)	20.5	(20.4–20.5)
0–4	‘09–10	331,452	(147,420–598,180)	5.8	(4.0–6.6)
0–4	‘10–11	898,531	(555,749–1,455,290)	29.2	(28.6–29.6)
**0**–**4**	**6**–**season total**	**2,843,053**	**(1,681,549**–**4,694,930)**	**15.7**	**(14.8**–**16.3)**
5–19	‘05–06	190,363	(102,511–334,862)	5.8	(5.2–6.2)
5–19	‘06–07	196,522	(108,191–339,485)	8.7	(8.8–8.7)
5–19	‘07–08	380,403	(234,028–622,070)	9.6	(9.5–9.6)
5–19	‘08–09	577,803	(360,831–936,431)	12.6	(12.6–12.6)
5–19	‘09–10	446,237	(208,880–910,431)	2.1	(1.5–2.8)
5–19	‘10–11	1,223,501	(754,823–1,993,709)	19.3	(19.1–19.6)
**5**–**19**	**6**–**season total**	**3,014,828**	**(1,769,264**–**5,136,988)**	**7.3**	**(6.7**–**7.8)**
20–64	‘05–06	280,160	(156,267–476,407)	7.8	(6.8–8.4)
20–64	‘06–07	259,640	(163,285–415,766)	10.5	(10.5–10.5)
20–64	‘07–08	892,219	(591,677–1,379,827)	11.2	(11.0–11.2)
20–64	‘08–09	448,906	(290,791–711,831)	12.6	(12.6–12.6)
20–64	‘09–10	490,901	(252,077–868,192)	2	(1.5–2.3)
20–64	‘10–11	1,635,765	(1,059,380–2,562,763)	13.7	(13.0–14.1)
**20**–**64**	**6**–**season total**	**4,007,591**	**(2,513,477**–**6,414,786)**	**7.4**	**(6.9**–**7.7)**
65+	‘05–06	548,659	(303,817–959,082)	17.4	(14.7–19.5)
65+	‘06–07	284,101	(175,790–458,439)	20.8	(20.7–21.0)
65+	‘07–08	1,307,926	(867,477–2,011,677)	20.4	(20.1–20.6)
65+	‘08–09	263,913	(162,638–427,898)	21.3	(21.2–21.3)
65+	‘09–10	55,178	(27,284–105,159)	2.4	(1.9–2.9)
65+	‘10–11	1,274,682	(500,228–2,597,822)	21.7	(12.9–28.2)
**65+**	**6**–**season total**	**3,734,459**	**(2,037,234**–**6,560,078)**	**18.4**	**(15.3**–**20.7)**
All ages	‘05–06	1,300,309	(718,135–2,262,138)	10.6	(9.3–11.6)
All ages	‘06–07	1,058,185	(637,990–1,735,867)	13.5	(13.5–13.6)
All ages	‘07–08	3,058,590	(1,990,600–4,783,002)	14.5	(14.4–14.6)
All ages	‘08–09	1,826,602	(1,148,959–2,934,230)	15.2	(15.1–15.2)
All ages	‘09–10	1,323,768	(635,661–2,481,963)	2.5	(1.8–3.0)
All ages	‘10–11	5,032,478	(2,870,179–8,609,584)	18.5	(16.0–20.3)
**All ages**	**6**–**season total**	**13,599,931**	**(8,001,525**–**22,806,782)**	**10.2**	**(9.2**–**10.9)**

**Table 3:** Estimated number of medically-attended (MA) influenza cases averted by vaccination, 2005/06–2010/11 influenza seasons (95% confidence interval in parentheses).

Age	Influenza season	Number of averted MA cases	
0–4	‘05–06	118,073	(66,081–214,000)
0–4	‘06–07	133,527	(80,762–226,718)
0–4	‘07–08	200,777	(124,972–336,387)
0–4	‘08–09	225,112	(141,780–376,297)
0–4	‘09–10	222,073	(98,566–401,175)
0–4	‘10–11	377,383	(234,575–634,163)
**0**–**4**	**6**–**season total**	**1,276,945**	**(746,735**–**2,188,741)**
5–19	‘05–06	79,952	(43,564–146,080)
5–19	‘06–07	82,539	(45,810–146,801)
5–19	‘07–08	159,769	(98,392–269,500)
5–19	‘08–09	242,677	(151,815–409,603)
5–19	‘09–10	227,581	(106,135–465,664)
5–19	‘10–11	513,870	(318,802–862,821)
**5**–**19**	**6**–**season total**	**1,306,389**	**(764,520**–**2,300,470)**
20–64	‘05–06	117,667	(66,351–207,808)
20–64	‘06–07	109,049	(69,214–181,233)
20–64	‘07–08	374,732	(249,901–603,264)
20–64	‘08–09	188,540	(121,493–311,448)
20–64	‘09–10	181,633	(92,823–322,744)
20–64	‘10–11	687,021	(447,295–1,119,398)
**20**–**64**	**6**–**season total**	**1,658,643**	**(1,047,078**–**2,745,894)**
65+	‘05–06	230,437	(128,709–417,093)
65+	‘06–07	119,322	(74,175–200,011)
65+	‘07–08	549,329	(367,817–883,761)
65+	‘08–09	110,844	(68,586–186,931)
65+	‘09–10	30,900	(15,220–59,141)
65+	‘10–11	535,366	(213,902–1,122,578)
**65+**	**6**–**season total**	**1,576,198**	**(868,410**–**2,869,516)**
All ages	‘05–06	546,130	(304,706–984,981)
All ages	‘06–07	444,438	(269,962–754,764)
All ages	‘07–08	1,284,608	(841,082–2,092,912)
All ages	‘08–09	767,173	(483,674–1,284,279)
All ages	‘09–10	662,187	(312,744–1,248,725)
All ages	‘10–11	2,113,641	(1,214,575–3,738,960)
**All ages**	**6**–**season total**	**5,818,175**	**(3,426,742**–**10,104,621)**

**Table 4:** Estimated number of influenza hospitalizations averted by vaccination, 2005/06–2010/11 influenza seasons (95% confidence interval in parentheses).

Age	Influenza season	Number of averted hospitalizations	
0–4	‘05–06	1,960	(1,084–3,429)
0–4	‘06–07	2,216	(1,330–3,640)
0–4	‘07–08	3,333	(2,073–5,364)
0–4	‘08–09	3,737	(2,333–5,982)
0–4	‘09–10	2,311	(1,028–4,170)
0–4	‘10–11	6,264	(3,874–10,146)
**0**–**4**	**6**–**season total**	**19,821**	**(11,723**–**32,731)**
5–19	‘05–06	522	(281–918)
5–19	‘06–07	539	(297–931)
5–19	‘07–08	1,043	(642–1,706)
5–19	‘08–09	1,584	(989–2,568)
5–19	‘09–10	1,224	(573–2,496)
5–19	‘10–11	3,355	(2,070–5,467)
**5**–**19**	**6**–**season total**	**8,266**	**(4,851**–**14,085)**
20–64	‘05–06	1,890	(1,054–3,215)
20–64	‘06–07	1,752	(1,102–2,805)
20–64	‘07–08	6,020	(3,992–9,311)
20–64	‘08–09	3,029	(1,962–4,803)
20–64	‘09–10	3,312	(1,701–5,858)
20–64	‘10–11	11,038	(7,148–17,293)
**20**–**64**	**6**–**season total**	**27,042**	**(16,960**–**43,285)**
65+	‘05–06	8,484	(4,698–14,830)
65+	‘06–07	4,393	(2,718–7,089)
65+	‘07–08	20,225	(13,414–31,107)
65+	‘08–09	4,081	(2,515–6,617)
65+	‘09–10	853	(422–1,626)
65+	‘10–11	19,711	(7,735–40,170)
**65+**	**6**–**season total**	**57,746**	**(31,502**–**101,439)**
All ages	‘05–06	12,856	(7,118–22,392)
All ages	‘06–07	8,900	(5,446–14,466)
All ages	‘07–08	30,621	(20,121–47,487)
All ages	‘08–09	12,431	(7,800–19,970)
All ages	‘09–10	7,700	(3,723–14,151)
All ages	‘10–11	40,367	(20,827–73,075)
**All ages**	**6**–**season total**	**112,875**	**(65,036**–**191,540)**

**Figure 1:**
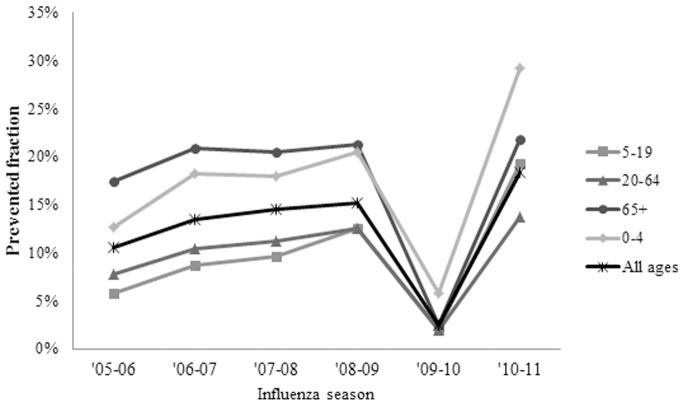
Prevented fraction of influenza cases, by age group, 2005/06–2010/11 influenza seasons.

**Figure 2:**
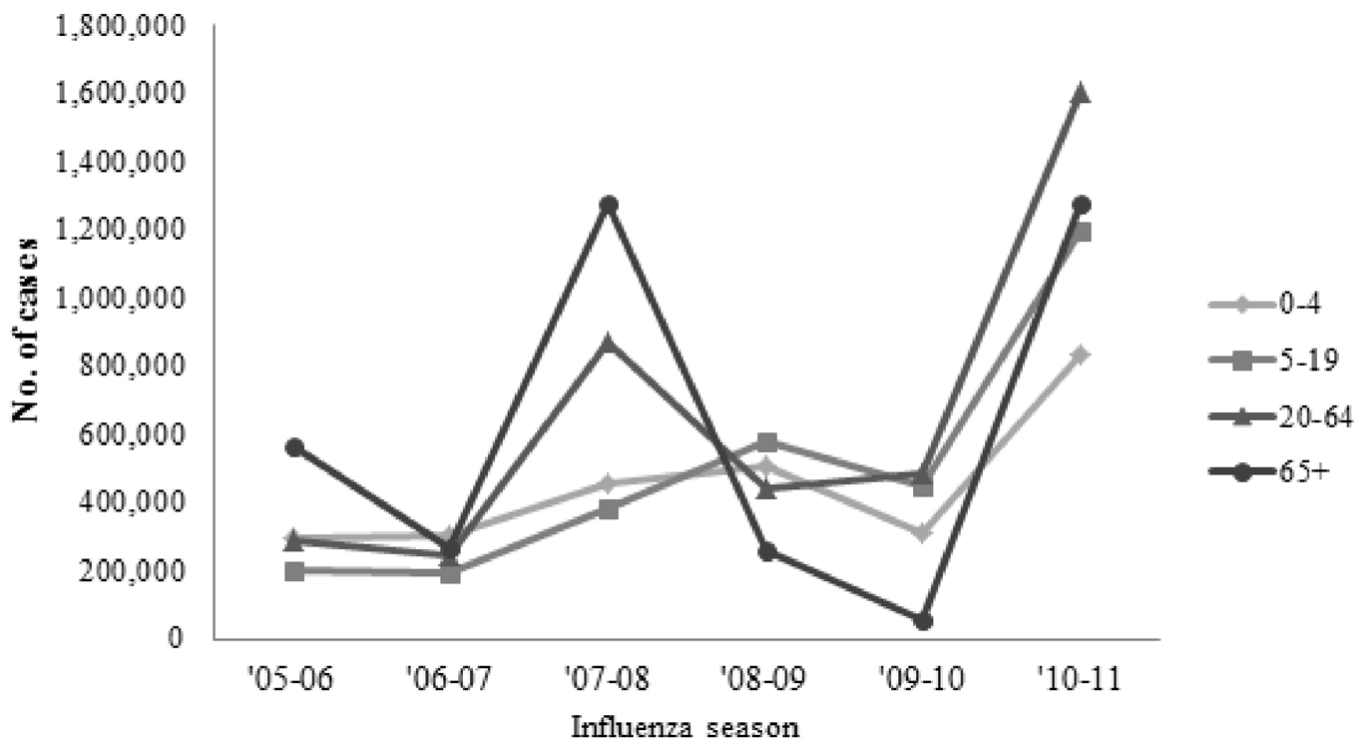
Number of averted influenza cases, by age group, 2005/06–2010/11 influenza seasons.

Before the 2009 pandemic, the prevented fraction increased over time from 10.6% (CI 9.3%–11.6%) in 2005–2006 to 15.2% (CI 15.1%–15.2%) in 2008–2009 (Figure 1) even as the number of averted outcomes fluctuated (Figure 2). The 2009 pandemic represented a break in this trend with a prevented fraction of 2.5% (CI 1.8%–3.0%). This decrease in the prevented fraction reflected the early timing of pandemic–associated disease relative to vaccine availability, despite a relatively high vaccine effectiveness conferred by the pandemic vaccine. Despite the lag in vaccination, the high attack rate associated with the 2009 H1N1 pandemic virus in younger populations resulted in an overall number of averted cases (1.3 (CI 0.6–2.5) million) that was comparable in magnitude relative to earlier seasons; in fact, the number of averted cases among adults aged 20–64 may have been higher during the pandemic than during the preceding season (Table 2).

Over the study period, the largest total number of averted outcomes occurred among adults 20–64 years of age, the largest age group in the study. Nevertheless, the 6-year average prevented fraction in this age group tended to be among the lowest at 7.4% (CI 6.9%–7.7%), similar to that in children aged 5–19 (Table 2). Before the 2009 pandemic, the prevented fractions were highest for persons 65 and older. During the 2009 pandemic, the highest prevented fraction occurred among children under 4 at 5.8% (CI 4.0%–6.6%), and remained higher than all other age groups in the post-pandemic 2010–2011 season as well, at 29.2% (CI 28.6%–29.6%) (Figure 1).

When estimating the impact of vaccination among the elderly, we accounted for the reduced effectiveness of the vaccine among the elderly by assuming that in seasons for which age-specific VE estimates were not available (all seasons except 2010/11), the VE for the elderly was a fraction the non-elderly VE. We set this fraction to 70% in the main analysis, and reflected the uncertainty of this assumption by performing a sensitivity analysis where we assumed that the elderly VE was, alternatively, 40% and 80% of the non-elderly VE. The results of this sensitivity analysis are shown as supplementary material in Table S1. We found that while the number of averted outcomes among the elderly was indeed higher at higher levels of VE, the overall number of averted outcomes in the entire population (all ages) was not substantially sensitive to variations in the elderly VE (Table S1), reflecting the relatively small proportion of elderly in the population.

## Discussion

While the vaccine produced benefits each season, the number of influenza-associated outcomes averted by vaccination fluctuated across age groups and seasons (Figure 2), reflecting the interplay of seasonal differences in vaccination coverage, vaccine effectiveness, and influenza attack rate. Of these impact determining factors, the attack rate is the only one not subject to human intervention, so from an impact evaluation standpoint it may be appropriate to assess impact net of its influence (seasons with higher attack rates could be associated with more averted cases simply because more people are susceptible to becoming ill, so that a greater number of illnesses could be prevented with the same level of vaccination and vaccine effectiveness). Calculating the prevented disease fraction instead of the number of averted cases can useful for describing the impact of vaccination programs because it controls for the relative severity of different seasons. The prevented fraction can also present a different pattern of impact over time than the number of averted outcomes alone – a pattern which more clearly reflects the benefits of increased vaccination coverage. The population-level benefits of increased vaccination become apparent when comparing the generally rising trend in vaccination coverage over time (Table 1) to the trend in the prevented fraction over time (Table 2) for each age group; this comparison shows that a greater fraction of disease was prevented as greater fractions of the population became vaccinated.

The prevented fraction during the 2009 pandemic was lower than during other seasons, highlighting the importance of the timing of vaccination relative to disease occurrence. While vaccination still prevented a considerable number of outcomes during the pandemic, the relatively late availability of the vaccine relative to the timing of pandemic disease in 2009–2010 resulted in a comparatively low prevented fraction during that year. Across age groups, the prevented fraction during the 2009 pandemic was highest among children aged 0–4, indicating that the priority schedule for vaccinating younger persons may have yielded a health benefit (Figure 1).

The increase in the prevented fraction during the 2010–2011 season relative to earlier seasons demonstrates how increases in vaccination coverage can drive substantial improvements in the impact of vaccination. Likely due to raised post-pandemic awareness about influenza, overall vaccination in 2010–2011 increased compared to both pandemic and pre-pandemic levels (Table 1). In 2010, a higher proportion of vaccinations also occurred earlier in time than during previous seasons, with relatively higher vaccination rates in the months of August and September [32]. The increases in vaccination coverage across all age groups that occurred in the season after the 2009 pandemic produced the largest vaccine impact observed over the 6-year study period both in terms of prevented fraction and number of averted cases (Figures 1 and 2).

Vaccination coverage tends to be lowest among adults aged 20–64, as many persons in this age group were not targeted for vaccination until the universal recommendations were issued in 2010. Because of the disproportionately large size of this population subgroup, increasing vaccination coverage among the nonelderly adults is likely to result in a large increase in the number of averted cases. While vaccination coverage among the elderly has been traditionally high (Table 1), the comparatively lower effectiveness of the influenza vaccine in this age group reduced the benefits that such high levels of vaccination could have otherwise produced. Improving vaccine effectiveness among the elderly would have an additional impact on averted influenza-associated outcomes, especially hospitalizations, which occur at much higher rates in this age group.

Our study is subject to a number of limitations, most of which are associated with the choice of input values used in the model. First, vaccine coverage data were obtained from NHIS and were based on self-report; some selection bias may remain even after weighting adjustments for survey nonresponse, and coverage data did not indicate the number of doses received. Second, due to lack of pre-pandemic data, we assumed that the hospitalization underreporting multiplier was the same during all nonpandemic seasons as during the 2009 pandemic. Since increased surveillance and awareness during the pandemic may have contributed to higher testing and higher hospitalization rates than during seasonal epidemics, using the underreporting multiplier calculated from 2009 data may result in conservatively low estimates of hospitalizations for non-pandemic seasons. However, recent studies have confirmed that these multipliers are similar between pandemic and non-pandemic periods [33]. Third, data on the variability of vaccine effectiveness among different age groups are limited. Similarly, data on the fraction of ILI that were medically attended in non-pandemic seasons were not available by season and age and thus a constant rate was assumed.

Vaccination against influenza has a substantial annual impact on the burden of disease in the United States. The study demonstrates that improvements in vaccination coverage among non-elderly persons and improvements in vaccine effectiveness among the elderly will lead to greater gains in program effectiveness. These data can be used to estimate the economic impact of vaccination programs on the national level. In addition, the model can estimate the effect of current policies on reducing disease among vulnerable groups such as pregnant women, and can be used to predict the impact of program improvements or alternative vaccination policies. Finally, because this study focuses on estimating the direct impact of vaccination only, further refinements that include estimation of the indirect impact of vaccination would yield a more complete estimate of the value of influenza vaccination programs in the United States.

## Supporting Information

Table S1 Total number of averted cases by influenza season: Sensitivity analysis on the assumption behind the elderly vaccine effectiveness adjustment.
(DOCX)

Appendix S1 **Technical appendix: Methodology description.**
(DOCX)
