# Supplementary material for: Influenza Illness and Hospitalizations Averted by Influenza Vaccination in the United States, 2005–2011
Source: PLoS One. 2013 Jun 19;8(6):e66312. doi: 10.1371/journal.pone.0066312 (PMC3686813; doi:10.1371/journal.pone.0066312)
Supplement: Table S1 — Total number of averted cases by influenza season: Sensitivity analysis on the assumption behind the elderly vaccine effectiveness adjustment. (DOCX) [file pone.0066312.s001.docx]

Table S1. Total number of averted cases by influenza season: Sensitivity analysis on the assumption behind the elderly vaccine effectiveness (VE) adjustment.

| Influenza season | Elderly VE is 70% of non-elderly VE (main analysis) |  | Elderly VE is 40% of non-elderly VE (sensitivity analysis) |  | Elderly VE is 80% of non-elderly VE (sensitivity analysis) |
| --- | --- | --- | --- | --- | --- |
| '05-06 | 1,341,149 |  | 1,082,679 |  | 1,433,883 |
| '06-07 | 1,017,304 |  | 891,624 |  | 1,063,234 |
| '07-08 | 2,982,843 |  | 2,388,738 |  | 3,199,269 |
| '08-09 | 1,793,145 |  | 1,670,858 |  | 1,837,963 |
| '09-10 | 1,299,587 |  | 1,275,643 |  | 1,307,749 |
| '10-11 | N/A |  | N/A |  | N/A |

Note: VE for 2010/11 season were available by age group. Therefore, no adjustment was applied to the elderly VE in that season.
