# Supplementary material for: Influenza Illness and Hospitalizations Averted by Influenza Vaccination in the United States, 2005–2011
Source: PLoS One. 2013 Jun 19;8(6):e66312. doi: 10.1371/journal.pone.0066312 (PMC3686813; doi:10.1371/journal.pone.0066312)
Supplement: Appendix S1 — Technical appendix: Methodology description. (DOCX) [file pone.0066312.s002.docx]

Appendix S1. Technical appendix: Methodology description

This appendix describes the methodology used to estimate averted burden in the manuscript.

Step A. Estimating the number of outcomes in the presence of vaccination

We first estimated the monthly number of hospitalizations that occurred each season using national EIP surveillance data corrected for under-ascertainment. We then estimated cases and medically-attended (MA) cases as simple multiples of the number of hospitalizations using available ratios of hospitalizations to cases and of cases to MA cases.

The number of hospitalizations that occurred among age group *a* in month *m* of season *s* was estimated by multiplying the total population in each age group by the EIP hospitalization rate for that month and the hospitalization underreporting multiplier of 2.7 (95% CI 1.7-4.5) from Reed et al. (2009) [1]:

*# Hospitalizations_sma_ =*

*= EIP hospitalization rate_sma_ * Hospitalization underreporting multiplier * Population_sma_* (1)

Where *s* denotes season, *m* denotes month, and *a* denotes age category.

The number of cases was estimated by multiplying the number of hospitalizations by the case-hospitalization ratio from Reed et al. (2009) [1]:

*# Cases_sma_ = #Hospitalizations_sma_ * Case-hospitalization ratio_a_* (2)

The number of medically attended (MA) cases was calculated as a fraction of the number of cases:

*# MA Cases_sma_ = # Cases_sma_ * MA fraction_sma_* (3)

Step B. Estimating the rate of influenza outcomes among susceptibles

For each month, the rate of an influenza-related outcome in the susceptible population was calculated as the number of each outcome calculated in step A divided by the total number of people estimated to be susceptible to influenza in that month. We refer to the number of influenza-susceptible individuals as the “population at risk”.

The population at risk was defined as those in each month not estimated to have previously had disease or been vaccinated, and thus varies over time depending on influenza incidence and vaccination coverage patterns. Before the start of each season, the initial population at risk equals the total U.S. population – in other words, everyone is assumed to be susceptible to influenza before immunity starts being acquired through either infection or vaccination. As a season progresses, the population at risk declines each month depending on how many people had already become sick or effectively vaccinated in previous months.

First, we estimated the population at risk at the beginning of each month. Then, we estimated the average population at risk for the month using the population at risk at the beginning of the month and adjusting it for the protective impact of vaccinations received during the month. In every month *m*, the population at risk at the beginning of the month is defined as the population at risk at the beginning of the prior month, *m*-1, after subtracting the number of cases and vaccinations received during the prior month:

*Beginning Population at Risk_sma|m=M_  =*

= (*Beginning Population at Risk_sma|m=M-1_ - Cases_sma|m=M-1_*) *** (1 *– IVC_sma|m=M-1_ * VE_sa_*) (4)

where *IVC_sma_* stands for incremental vaccination coverage – i.e., the proportion of the population vaccinated during month *m*. Eq. 4 removes from each month’s initial pool of susceptibles all individuals who acquire immunity during that month due to either illness or vaccination; the number of cases are subtracted *before* factoring in the protective effect of vaccination in order to avoid double-counting the impact of vaccination among those who have already been ill. In Eq. 4, the incremental monthly vaccination rate *IVC_sma_* was multiplied by the season’s vaccine effectiveness rate, *VE_sa_*, to account for the fact that not all vaccinations translate into full protection. Furthermore, throughout this study, the incremental vaccination coverage rate *IVC_sma_* was adjusted to allow for a two-week lag between vaccination and immunogenic response, as follows. Data on vaccination coverage were available as monthly figures only, so in order to account for a two-week lag between the time when a vaccine was administered and the time it becomes protective we assumed that vaccinations were administered uniformly throughout each month. Using that assumption, we defined each month’s lag-adjusted *IVC_sma_* as half of the administered incremental vaccination coverage from the prior month and half of the administered incremental vaccination coverage from the current month:

*IVC_sma|m=M_* = 0.5**IVC administered_sma|m=M-1_ +* 0.5**IVC administered_sma|m=M_* (5)

In other words, for each current month, vaccinations administered in the first half of the month become protective in the second half, while vaccinations administered in the second half of the prior month become protective in the first half of the current month.

Once we estimated the population at risk at the beginning of each month (Eq. 4), we used these estimates to calculate the average population at risk during each month. The average population at risk in each month is the pool of susceptibles from which cases occur throughout the month; in later steps of the model it would be used as a denominator to determine the monthly rate of illness. The average population at risk in each month is not the same as the population at risk at the beginning of the month described by Eq. 4 because the population at risk decreases over time in response to vaccinations that occur throughout the month. We follow the method outlined in Collet (1994, p.17) to estimate the average monthly population at risk given a certain rate of vaccination during the month [2].

*Average Population at Risk_sma_=*

*=* (*Beginning Population at Risk_sma_*) *** (1 *– VE_sa_ * IVC_sma_* *0.5) (6)

Eq. 6 discounts the population at risk at the beginning of each month by using an actuarially-adjusted monthly protective effect of vaccination where the incremental monthly vaccination *IVC_sma_* was assumed to be distributed uniformly throughout each month.

The average monthly population at risk from Eq. 6 was the basis for estimating the rates at which influenza outcomes occurred in the susceptible population throughout a season. We called these monthly rates “outcome rates”, and estimated them by age group for each influenza outcome as follows:

*Rate of influenza illness_sma_ = # Cases_sma_ / Average Population at Risk_sma_* (7)

*Rate of MA influenza illness_sma_ = # MA cases_sma_ / Average Population at Risk_sma_*  (8)

*Rate of influenza hospitalization_sma_ =*

*= # Hospitalizations_sma_ / Average Population at Risk_sma_* (9)

In the next step of the model, we used these rates to project the number of outcomes in the absence of vaccination by applying them to the estimated population at risk in the absence of vaccination.

Step C. Estimating the number of outcomes in the absence of vaccination

To estimate the number of outcomes in the absence of vaccination, we first projected what the population at risk would have been without adjusting for vaccine-acquired immunity while still adjusting for disease-acquired immunity. We then multiplied the monthly rates among the susceptible population for each outcome (as calculated in Step B) to the monthly population at risk in the absence of vaccination to estimate the projected number of outcomes that would have occurred in the absence of vaccination.

The population at risk in the absence of vaccination in the first month of each season, *m*=1, is simply the total US population, by age group, since no disease or vaccination is assumed to have occurred prior to that time.

*Population at risk w/o vax_sma|m=1_ = Total Population_sma|m=1_* (10)

Then, the number of cases in the absence of vaccination that occur in the first month is equal to the product of the first month’s population at risk (also equal to the total population since *m*=1) and the month’s rate of influenza illness from Eq. 7:

*# Cases w/o vax _sma|m=1_  = Rate of influenza illness_sma|m=1_ * Total Population_sma|m=1_* (11)

For all subsequent months, denoted as *m*=M, we estimated each month’s population at risk in the absence of vaccination as the corresponding population at risk from the prior month, reduced by the number of cases that would have occurred in the prior month in the absence of vaccination. Therefore, each month’s estimated number of cases in the absence of vaccination was used to determine the following month’s population at risk in the absence of vaccination. Each month’s number of cases in the absence of vaccination was estimated as the product of the month’s population at risk in the absence of vaccination and the month’s rate of influenza illness from Eq. 7, as follows:

*Population at risk w/o vax_sma|m=M_ =*

*= Population at risk w/o vax_sma|m=M-1_ – Cases w/o vax_sma|m=M-1_*  (12)

*# Cases w/o vax_sma|m=M_  =*

*= Population at risk w/o vax_sma|m=M_ * Rate of influenza illness_sma|m=M_* (13)

Once the population at risk and the number of cases in the absence of vaccination was estimated for each month, we estimated the number of other outcomes by applying the outcome-specific rates to the population at risk in the absence of vaccination:

*# MA cases w/o vax_sma_ = Rate of MA influenza illness_sma_ * Population at risk w/o vax_sma_* (14)

*# Hospitalizations w/o vax_sma_ =*

*Rate of influenza hospitalization_sma_ * Population at risk w/o vax_sma_* (15)

Finally, we took the difference between the observed number of outcomes with vaccination from Step A and the estimated number of outcomes without vaccination from Step C to estimate the disease burden averted by vaccination.

To calculate confidence intervals, we used a Monte Carlo algorithm drawing values from sampling distribution of the input variables used in the calculations. Sampled values were used in the full series of calculations described, in each of 5,000 iterations. Confidence intervals represent the 2.5th and 97.5th percentile of the resulting empirical distribution. The sampling distributions used for each of the model inputs were as follows: for vaccine coverage, normal distribution fit to the monthly point estimate and standard error; for vaccine effectiveness, normal distribution fit to the annual pooled point estimate and weighted standard error; for the medically-attended fraction, normal distribution fit to the annual point estimate and standard error; for the rate of hospitalization, Poisson distribution fit to the monthly count of reported hospitalizations, for the hospitalization underreporting multiplier, triangular distribution centered on the point estimate, between the minimum and maximum values. These analyses were performed in SAS version 9.3 (SAS Institute, Cary, NC).

References:

1. Reed C, Anjulo FJ, Swerdlow DL, Lipsitch M, Meltzer MI et al. (2009) Estimates of the prevalence of pandemic (H1N1) 2009, United States, April-July 2009. Emerging Infectious Diseases 15(12):2004-2007.
2. Collet D (1994) Modelling survival data in medical research. Chapman & Hall, London, UK.
